# Sepsis survivors and caregivers perspectives on post–acute rehabilitation and aftercare in the first year after sepsis in Germany

**DOI:** 10.3389/fmed.2023.1137027

**Published:** 2023-04-11

**Authors:** Sebastian Born, Claudia Matthäus-Krämer, Anna Bichmann, Hannah-Sophia Boltz, Marlene Esch, Luisa Heydt, Stefan Sell, Kathleen Streich, André Scherag, Konrad Reinhart, Christiane S. Hartog, Carolin Fleischmann-Struzek

**Affiliations:** ^1^Institute of Infectious Diseases and Infection Control, Jena University Hospital, Jena, Germany; ^2^Center for Sepsis Control and Care, Jena University Hospital, Jena, Germany; ^3^Department of Anesthesiology and Operative Intensive Care, Charité-Universitätsmedizin Berlin, Berlin, Germany; ^4^Institute of Medical Statistics, Computer and Data Sciences, Jena University Hospital, Jena, Germany; ^5^Klinik Bavaria, Kreischa, Germany

**Keywords:** sepsis, rehabilitation, aftercare, post-sepsis-syndrome, survivors

## Abstract

**Background:**

Sepsis survivors often suffer from new morbidities. Current rehabilitation therapies are not tailored to their specific needs. The perspective of sepsis survivors and their caregivers on rehabilitation and aftercare is insufficiently understood. We aimed to assess how sepsis survivors in Germany rated the suitability, extent and satisfaction with rehabilitation therapies that they underwent in the year following the acute sepsis episode.

**Methods:**

Prospective mixed-methods, multicenter study among a cohort of adult ICU-treated sepsis survivors and their caregivers. Interviews were conducted 6 and 12 months after ICU discharge by telephone and comprised closed as well as open-ended questions. Primary outcomes were the utilization and patient satisfaction with inpatient and outpatient rehabilitation and post-sepsis aftercare in general. Open-ended questions were analyzed according to the principles of content analysis.

**Results:**

Foun hundred interviews were performed with 287 patients and/or relatives. At 6 months after sepsis, 85.0% of survivors had applied for and 70.0% had undergone rehabilitation. Among these, 97% received physical therapy, but only a minority reported therapies for specific ailments including pain, weaning from mechanical ventilation, cognitive deficits of fatigue. Survivors were moderately satisfied with the suitability, extent, and overall results of received therapies and perceived deficits in the timeliness, accessibility, and specificity of therapies as well as deficits in the structural support frameworks and patient education.

**Conclusion:**

From the perspective of survivors who undergo rehabilitation, therapies should already begin in hospital, be more appropriate for their specific ailments and include better patient and caregiver education. The general aftercare and structural support framework should be improved.

## Highlights

- Current inpatient rehabilitation for sepsis survivors in Germany is mostly provided in neurological rehabilitation facilities.
- Therapies mostly focus on physical impairments, whereas less than a quarter of therapies address other ailments, such as pain, weaning from mechanical ventilation, psychological ailments or fatigue.
- Sepsis survivors perceive the suitability, extent, and outcome of rehabilitation therapies as moderately positive.
- Survivors and caregivers describe unmet needs regarding sepsis knowledge, timely rehabilitation, and structural support frameworks for aftercare.

## Introduction

Sepsis survivors frequently suffer from long-term sequelae and a reduced health-related quality of life (1, 2). Among older US-Americans, 59.3% of sepsis survivors experienced a worsened cognitive and/or physical function after sepsis (1). An analysis of nationwide German health claims data found that 74.3% of sepsis survivors suffered from new diagnoses and 31.5% were newly dependent on nursing care (3). Many survivors are unable to return to work and require assistance in the activities of daily living (4). Effective treatments and standards for rehabilitation to mitigate long-term impairments are still scarce (5). This likely contributes to the many survivors reporting dissatisfaction with the care and support services provided after hospital discharge as pointed out in an international survey amongst 1,731 respondents from 41 countries (6). Improvements of post-sepsis care and the development of specialized follow-up programs for sepsis survivors are therefore urgently demanded by the World Health Organization and the International Sepsis Forum (5, 7).

Rehabilitation programs are one important measure of post-sepsis care. They can help survivors to regain their functional independence and to reintegrate into normal life as fully as possible - one of the most important domains of their health-related quality of life (8). In the German health care system, patients can apply for medical rehabilitation at their health insurance that determines its type, duration, scope, starting date and implementation. Only rehabilitation institutions which have a service provision contract with health insurance can provide inpatient services (9). Inpatient rehabilitations usually have a duration of 3–4 weeks and provide specialized therapies depending on the subspecialty of the rehabilitation facility. Due to lack of specialized post-sepsis rehabilitation facilities, sepsis survivors are usually treated in neurological, cardiac or orthopedic rehabilitation facilities. In addition, patients have the option of receiving outpatient rehabilitation treatments, such as speech and language, physical or occupational therapy with a varying frequency. To date, we lack knowledge about the current rehabilitation treatment practices after sepsis and how they would best address the needs of sepsis survivors and their caregivers.

We therefore aimed to (i) assess the use of and satisfaction with inpatient and outpatient rehabilitation of sepsis survivors, as well as (ii) to describe the unmet needs of survivors and caregivers.

## Materials and methods

This prospective mixed-methods study was approved by the Institutional Review Boards of the Friedrich-Schiller University Jena (2018-1223-Bef) and Charité Universitätsmedizin Berlin (EA4/060/19).

### Patient cohort

All adult Intensive Care Unit (ICU) patients with sepsis were recruited between January 2019 and September 2020 from two tertiary care ICUs (medical and surgical specialties, Jena University Hospital and Charité Universitätsmedizin Berlin). Eligible patients were identified from daily screening of the electronic health records and the database of the “Mid German Sepsis cohort” (MSC). Inclusion criteria were age ≥18 years and the diagnosis of sepsis requiring ICU admission. Sepsis was defined by organ dysfunction due to infection (10). The criteria used are reported in Supplementary material S1. Furthermore, patients enrolled in the MSC were invited to participate in our survey study. The MSC is a large prospective cohort of sepsis survivors, who were consecutively enrolled into the MSC after receiving sepsis treatment on one of the five ICUs in Mid Germany (University Hospitals Halle, Leipzig, Jena, Helios Hospital Erfurt, Zentralklinik Bad Berka) between April 2016 and November 2018. For details on the MSC, we refer to the study protocol and cohort profile (11, 12). All patients who were not fluent in German were excluded from the study. To participate in the study, written informed consent by the patient or a legal guardian was required. Each patient was contacted at least twice after hospital discharge with an invitation to participate.

### Data collection

Demographics and clinical characteristics of participants were extracted from electronic health records by trained study nurses (Jena University Hospital, MSC patients) or by database inquiries (Charité Berlin). Interviews were scheduled within a six-week time frame after 6 months (follow-up 1) and 12 months (follow-up 2) post discharge from the ICU. Interviews were conducted with patients, relatives or legal representatives by trained physicians or students by telephone and comprised closed as well as open-ended questions (Supplementary material S2). Answers were documented immediately during telephone interviews. Patients who missed the first follow-up interview were eligible to participate in the second follow-up interview.

### Outcomes

We investigated the utilization and patient satisfaction with inpatient and outpatient rehabilitation and post-sepsis aftercare in general. We assessed the range of subspecialties of rehabilitation facilities study used by our study participants, which therapies they received during their rehabilitation and how these addressed the needs of their subjective physical, psychological or cognitive impairments. The satisfaction with rehabilitation and aftercare was assessed with three items. Patients rated (1) suitability of the rehabilitation therapy, i.e., whether overall rehabilitation was suited to their impairments, (2) extent, i.e., whether the extent of therapy received was sufficient and (3) satisfaction with the outcome of rehabilitation. Answers were assessed by a 4-point-Likert-Scale ranging from 1 = ‘does not apply’ to 4 = ‘does apply’. The ratings were obtained for aftercare and rehabilitation in general, as well as for each specific impairment for which a rehabilitation therapy was performed. We assessed predictors of satisfaction with aftercare at 12 months post-sepsis among patients that received rehabilitation. Potential predictors were identified by literature search and included extent and suitability of aftercare, age, sex, employment state, number of comorbidities according to the Charlson Comorbidity Index (CCI), maximum Sepsis-related organ failure assessment score (SOFA score), hospital length of stay and use of outpatient therapies. For the CCI, only 17 of the 19 comorbidities were used to calculate the score, as in a subsample no data were available for leukemia and malignant lymphoma. In order to check whether the score of the truncated form was reliable, the correlation between the score of the truncated CCI and the complete CCI was calculated for the subsample with data for all 19 comorbidities (*r* = 0.99). Furthermore, we investigated associations between health-related quality of life as assessed by the EQ-5D-3L (13) and the satisfaction with aftercare, and nursing care dependency and satisfaction with aftercare at 12 months post-sepsis. Unmet needs of patients and their caregivers were assessed by open-ended questions.

### Analyses

Continuous data are reported as means with standard deviations and medians with interquartile ranges [IQR]. For categorical variables, we calculated proportions. To identify predictors of the patients’ satisfaction at 12 months post-sepsis, we performed a multiple linear regression analysis in R (14) using the R package lavaan (15). Standardized regression coefficients (β) from these analyses were reported with 95% confidence intervals and *p*-values. For the full model, we used *R*^2^ as indicator for explained variance. We applied a significance level of *α* = 0.05. Full Information Maximum Likelihood [FIML, (16)] was used to handle missing data in the regression analysis. To investigate the association between health-related quality of life assessed by the EQ-5D-3L and the satisfaction with aftercare 12 months post-sepsis we calculated Pearson’s correlation. For the association between nursing care dependency and satisfaction with aftercare 12 months post-sepsis a two-sample *t*-test was conducted. Unmet needs of survivors and caregivers were collected by using open-ended questions. Based on these, replies were analyzed according to the principles of content analysis (17) by two independent social scientists using the MAXQDA software (18). If one interviewee named more than one complemented deficit or wish, only one was counted to avoid double counting.

## Results

**Figure 1:**
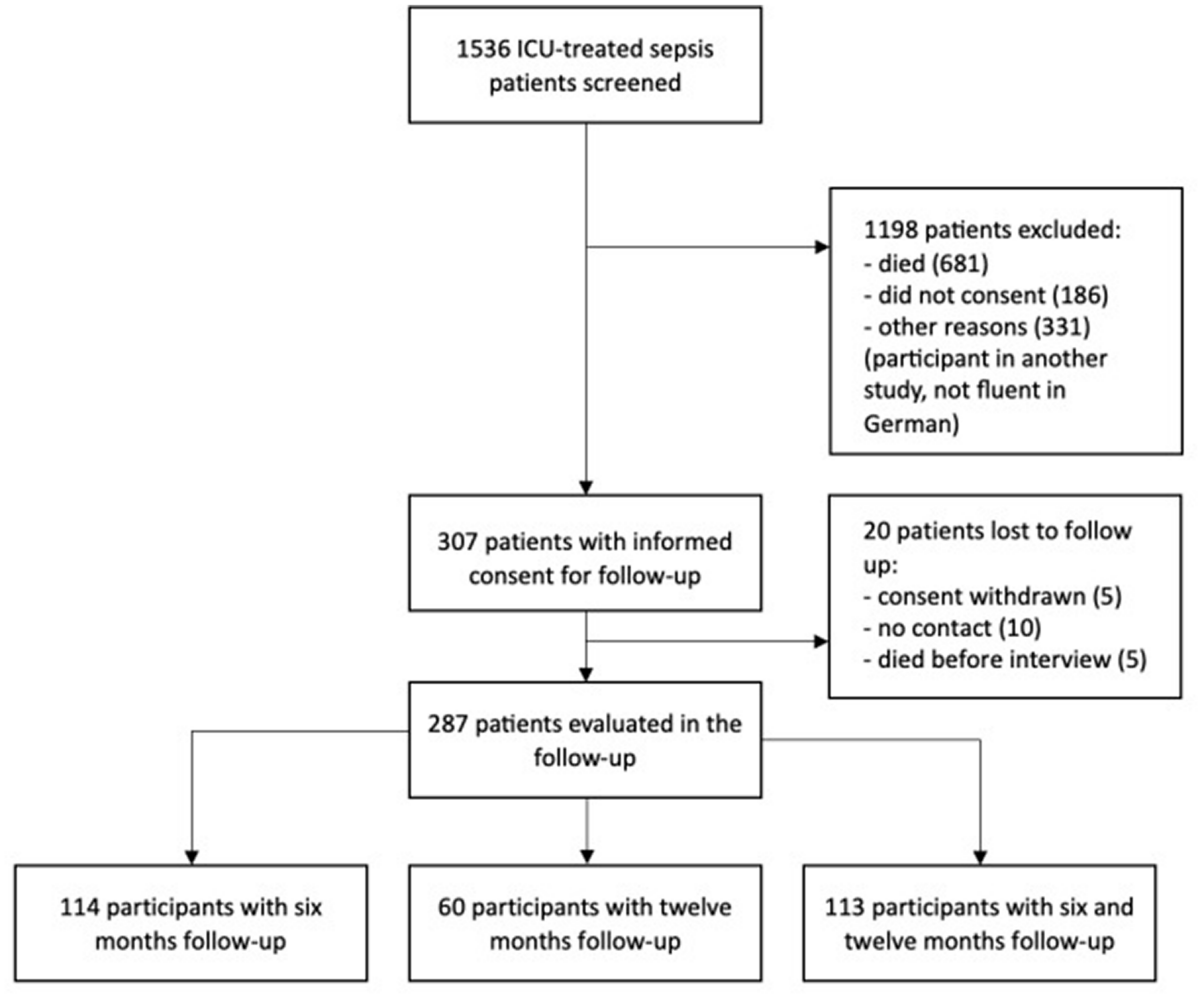
Flow of patient inclusion.

Out of 1,536 screened patients with ICU treated sepsis, 855 survived the hospitalization. Among them, 307 provided informed consent to participate in our study (Figure 1). 20 patients were lost to follow-up. In the remaining 287 patients, 400 interviews were performed with patients and/or relatives (227 at 6 months and 173 at 12 months after ICU discharge). 113 patients were interviewed consecutively at 6 and 12 months post ICU discharge, while 114 and 60 patients participated in either 6- or 12-months interviews, respectively. 68.8% of interviews were conducted with the patients, 21.3% with their caregivers and 10.1% with both.

Patients’ demographics and post-sepsis characteristics are provided in Tables 1, 2. The median age of participants was 65 (IQR 17) years, 67.2% were male. Half of patients were treated for septic shock (50.2%). The median maximum SOFA score was 11 (IQR 5), and 88.2% of patients received mechanical ventilation (Table 1). At 6 (and 12 months) after hospital discharge, 85.0% (89.6%) of patients had returned home while 7.9% (5.8%) were living in nursing homes and 48.9% (49.7%) of patients were dependent on formal or informal nursing care (Table 2). At 6 months, the mean EQ-5D-3L visual analogue scale (VAS) was 55.0 ± 24.0 (standard deviation, SD) and the mean utility score 0.62 ± 0.35 (SD). At 12 months, mean EQ-5D-3L VAS and utility score remained unchanged [55.3 ± 25.5 (SD) and 0.61 ± 0.37, respectively].

**Table 1:** Patient demographics and clinical features.

	At least one interview (*n* = 287)
I. Patient demographics	
Age, mean (SD), median (IQR)	69.9 (13.8), 65 (17)
Female sex, *n* (%)	94 (32.8)
Patients with pre-existing comorbidities, *n* (%)	238 (82.9)
CCI, unweighted, mean (SD), median (IQR)	1.79 (1.36), 2 (2)
Patients with pre-existing formal nursing care level, *n* (%)	48 (16.7)
Patients with prior employment, *n* (%)	94 (32.8)
II. Clinical features	
Focus of infection, *n* (%)	
Respiratory	73 (50.3)^b^
Thoracic	9 (6.2)^b^
Abdominal	36 (24.8)^b^
Cardiovascular	14 (9.7)^b^
Genitourinary	10 (6.9)^b^
Device-related	0 (0.0)^b^
Central nervous	2 (1.4)^b^
Primary sepsis	14 (9.7)^b^
Surgical site infection	2 (1.4)^b^
Bone/Soft tissue	8 (5.5)^b^
Occurrence of septic shock, *n* (%)	119 (82.1)^b^
Max. SOFA score, mean (SD), median (IQR)	11.1 (3.6), 11 (5.3)
Requirement of mechanical ventilation, *n* (%)	253 (88.2)
Requirement of renal replacement therapy, *n* (%)	89 (31.0)
ICU length of stay, mean (SD), median (IQR)	20.4 (22.9), 14 (22)
Hospital length of stay, mean (SD), median (IQR)	44 (41.2), 33 (27)

^a^New care level according to German care level system or new nursing home residence, ranging from grade 1 (“Little impairment of independence”) to grade 5 (“Hardship cases”). ^b^Data were only available for patients recruited in the Jena University Hospital (*n* = 145).

**Table 2:** Post-sepsis characteristics of survivors.

	At 6 months (*n* = 227)	At 12 months (*n* = 173)
I. Current living situation		
At home, *n* (%)	193 (85.0)	155 (89.6)
Nursing care residence, *n* (%)	18 (7.9)	10 (5.8)
Still hospitalized/in rehabilitation, *n* (%)	10 (4.4)	5 (2.9)
Other place, *n* (%)	6 (2.6)	3 (1.7)
II. Dependence on nursing care		
Patients with any reported nursing care dependency, *n* (%)	111 (48.9)	86 (49.7)
Care provided by, *n* (%)		
partner	58 (52.3)	44 (51.2)
other family	34 (30.6)	31 (36.1)
friends	0 (0.0)	3 (3.5)
nursing care service	47 (42.3)	43 (50.0)
other	10 (9.1)	3 (3.5)
Patients with formal nursing care level, *n* (%)	105 (46.3)	85 (49.1)
III. Inpatient rehabilitation therapies		
Patients who applied for rehabilitation, *n* (%)	183 (80.6)	147 (85.0)
Rehabilitation was applied for: *n* (%)		
Physical impairments	167 (91.3)	128 (87.1)
Cognitive impairments	24 (13.1)	24 (16.3)
Psychological impairments	21 (11.5)	15 (10.2)
Acute or chronic pain	12 (6.6)	13 (8.8)
Difficulties with normal activities/fatigue	45 (24.6)	24 (16.3)
Weaning from mechanical ventilation	20 (10.9)	16 (10.8)
Other impairments	34 (18.6)	25 (17.0)
Status of application,		
Rejected applications, *n* (%)	7 (3.8)	10 (6.9)
Accepted applications, *n* (%)	162 (88.5)	135 (92.5)
Pending applications, *n* (%)	14 (7.7)	1 (0.7)
Patients with at least 1 inpatient rehabilitation therapy, *n* (%)	145 (63.9)	121 (70.0)
Number of inpatient rehabilitation therapies, mean (SD), median (IQR)	0.7 (0.6), 1 (1)	0.8 (0.6), 1 (1)
IV. Outpatient rehabilitation therapies		
Patients with at least 1 outpatient rehabilitation therapy, *n* (%)	118 (52.0)	92 (53.2)
Therapies, *n* (%)		
Speech and language therapy	14 (12.0)	16 (17.4)
Physical therapy	102 (87.2)	77 (83.7)
Occupational therapy	22 (18.8)	26 (28.3)
Rehabilitation sports	11 (9.4)	13 (14.1)
Psychotherapy	8 (6.8)	5 (5.4)
Pain therapy	3 (2.6)	1 (1.1)
Wound management	12 (10.3)	5 (5.4)
Memory training	3 (2.6)	2 (2.2)
Other outpatient therapies	15 (12.8)	7 (7.6)
Number of outpatient rehabilitation therapies, mean (SD), median (IQR)	1.6 (1.0), 1 (1)	1.7 (0.9), 1 (1)
Duration of outpatient rehabilitation therapies in days, mean (SD), median (IQR)	15.7 (19.9),10.5 (15.8)	43.1 (62.2), 20 (46)

### Rehabilitation: Utilization and patient satisfaction

At 6 months post-sepsis, 80.6% (*n* = 183) of survivors had applied for inpatient rehabilitation while 63.9% (*n* = 145) had actually received inpatient rehabilitation. At 12 months post-sepsis, these proportions increased to 85.0% (application) and 70.0% (completed rehabilitation). Overall, 6.9% of rehabilitation applications were rejected by health insurance providers at 12 months post-sepsis.

**Figure 2:**
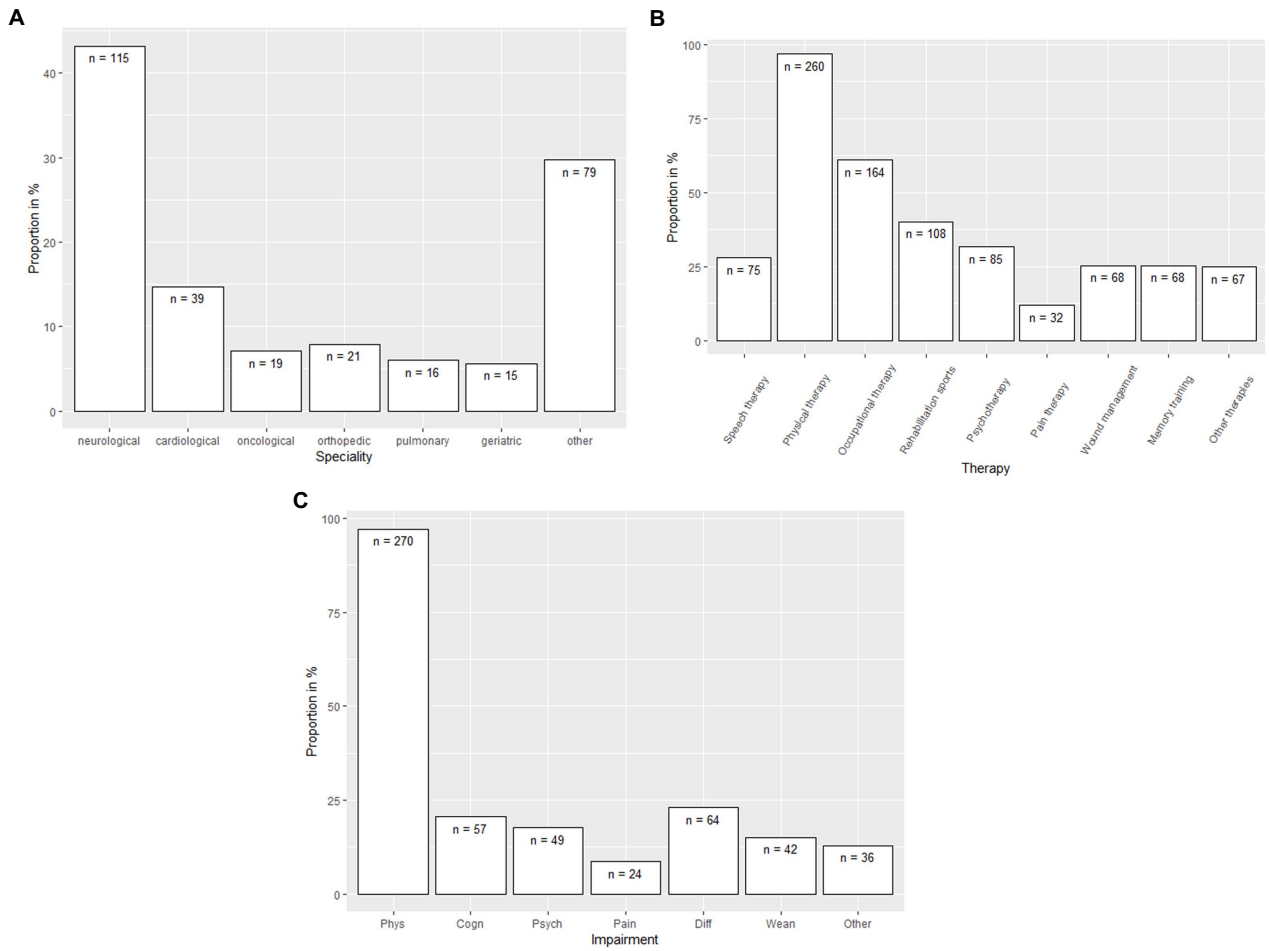
**(A)** Specialties of inpatient rehabilitation utilized by sepsis survivors (combined for 6 and 12 months interview). **(B)** Therapies received in rehabilitation by sepsis survivors (combined for 6 and 12 months interview). **(C)** Impairments of sepsis survivors addressed during the rehabilitation (combined for 6 and 12 months interview).

Patients mostly applied for rehabilitation for physical impairments (Table 2). Patients who had received inpatient rehabilitation were most often treated in rehabilitation facilities with neurological focus (43.2%, Figure 2A). Common rehabilitation therapies included: 96.7% physical therapy, 60.7% occupational therapy, and 40.1% rehabilitation sports (Figure 2B). Figure 2C shows impairments addressed by rehabilitation therapies. Rehabilitation most often addressed survivors’ physical impairments (97.3%), and less often other impairments, ranging from pain (8.6%) to difficulties with normal activities/fatigue (23%). Of note, psychological problems (17.6%) and weaning from ventilation (12.9%) were rarely addressed.

**Figure 3:**
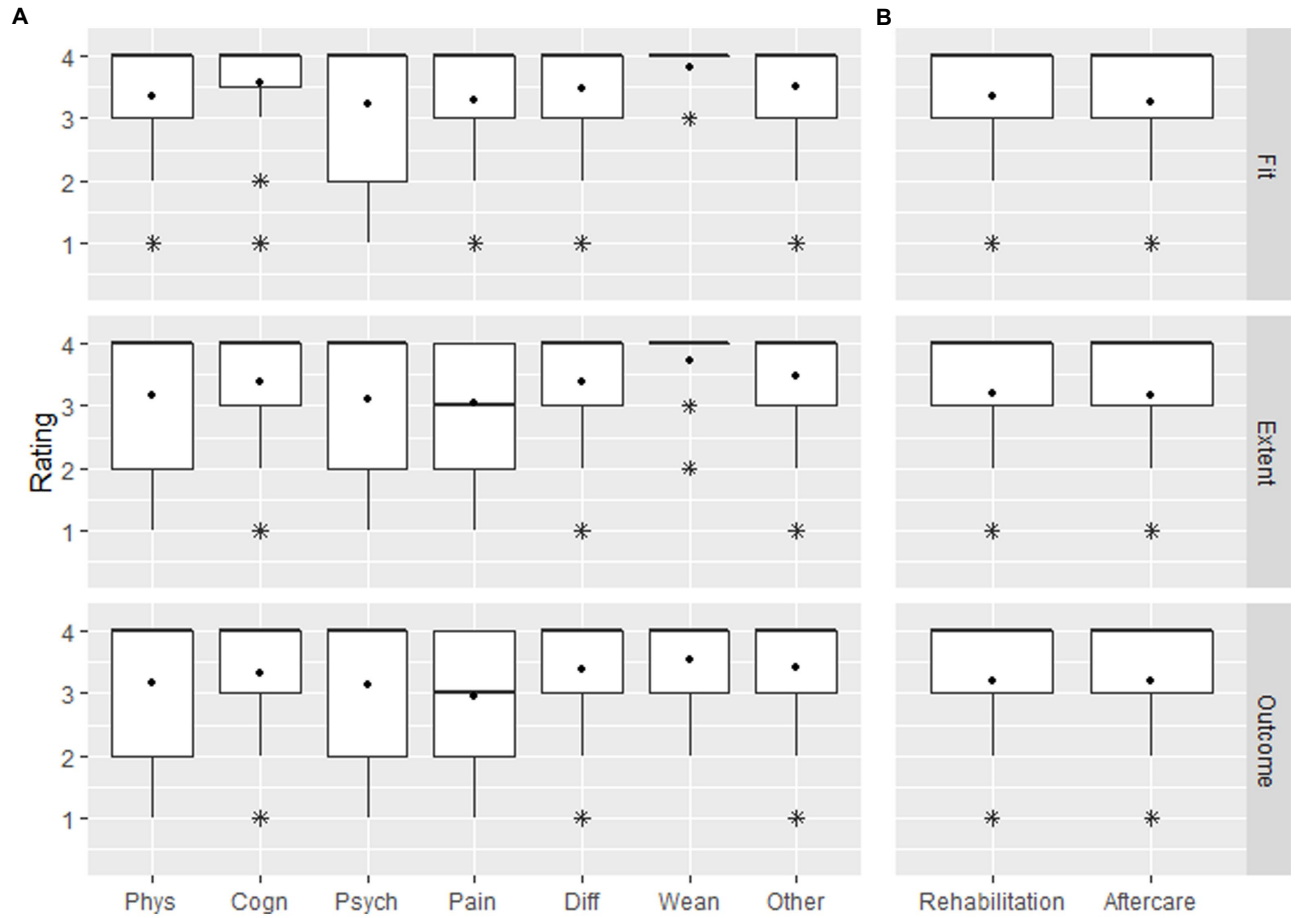
Rating of satisfaction with fit, extent and outcome of **(A)** rehabilitation by impairment and **(B)** rehabilitation and aftercare in general (combined for 6 and 12 months interview).

Overall rating of suitability, extent and outcome of rehabilitation therapies received at 6 and 12 months was moderately positive (mean 3.2 [SD 1.1] to 3.4 [SD 1.0] on a scale from 1 [negative] to 4 [positive]; Figure 3). Respondents also rated therapies for specific ailments moderately positive (mean between 3.0 and 3.8), with the highest rating for weaning from mechanical ventilation (mean 3.8 [SD 0.4] to 3.6 [SD 0.7] and the lowest rating for pain and psychological impairments (mean 3.0 to 3.3, Figure 3). Likewise, rating for inpatient and outpatient aftercare in general was similar (mean 3.3 [SD 1.0], to 3.2 [SD 1.0], Figure 3).

At 6 months (and 12 months) post-sepsis, 51.5% (52.6%) of survivors received outpatient rehabilitation therapies, respectively. Physical therapy (44.9%), occupational therapy (15.0%) and speech and language therapy (9.2%) were most common therapies. The median duration of these therapies was 20 IQR = 46 days.

### Satisfaction with aftercare

Average rating of suitability, extent, and satisfaction with aftercare at 6 and 12 months was also moderately positive (mean 3.1–3.3, Figure 3). In the multiple linear regression, we found that suitability and extent of aftercare were significantly associated with satisfaction with aftercare at 12 months post-sepsis (*β* = 0.149, *p* = 0.008, and *β* = 0.827, *p* < 0.0001, respectively). Age, gender, the number of comorbidities according to Charlson Comorbidity Index, employment state, maximum SOFA score, hospital length of stay and the provision of outpatient rehabilitation therapies were not associated with the patients’ satisfaction with aftercare (Table 3). The model explained 92% of the variance of 12-months satisfaction with aftercare (*R*^2^ = 0.92). Moreover, 12-months satisfaction with aftercare was slightly positively correlated with EQ-5D-3L VAS at that time point (*r* = 0.28). We found that the satisfaction with aftercare did not differ significantly between groups with (3.1 ± 1.1 [SD]) and without (3.2 ± 1.1 [SD]) need for formal or informal nursing care (*p* = 0.701). Average satisfaction with aftercare were similar at 6 and 12 months in patients that participated in both interviews (3.2 ± 1.1 and 3.1 ± 1.1, respectively).

**Table 3:** Standardized regression coefficients, *p*-values and 95%-confidence intervals from the multiple linear regression analyses for the outcome satisfaction with aftercare at 12 months after ICU discharge (*n* = 122).

	*β*	SE	*p*-value	95% – Confidence Interval	
				Lower bound	Upper bound
Age	−0.023	0.029	0.437	−0.08	0.034
Gender	0.023	0.028	0.406	−0.031	0.077
CCI	−0.007	0.028	0.796	−0.063	0.048
Employment state	−0.016	0.029	0.582	−0.074	0.042
Maximum SOFA Score	0.001	0.028	0.963	−0.054	0.056
Length of Hospital Stay	−0.012	0.03	0.685	−0.07	0.046
Use of Outpatient Therapies	0.012	0.03	0.689	−0.047	0.071
Suitability of Aftercare	0.149	0.056	0.008	0.039	0.259
Extent of Aftercare	0.827	0.055	< 0.001	0.718	0.935

CCI, Charlson Comorbidity Index was calculated based on 17 comorbidities.

### Unmet needs of survivors and caregivers

**Figure 4:**
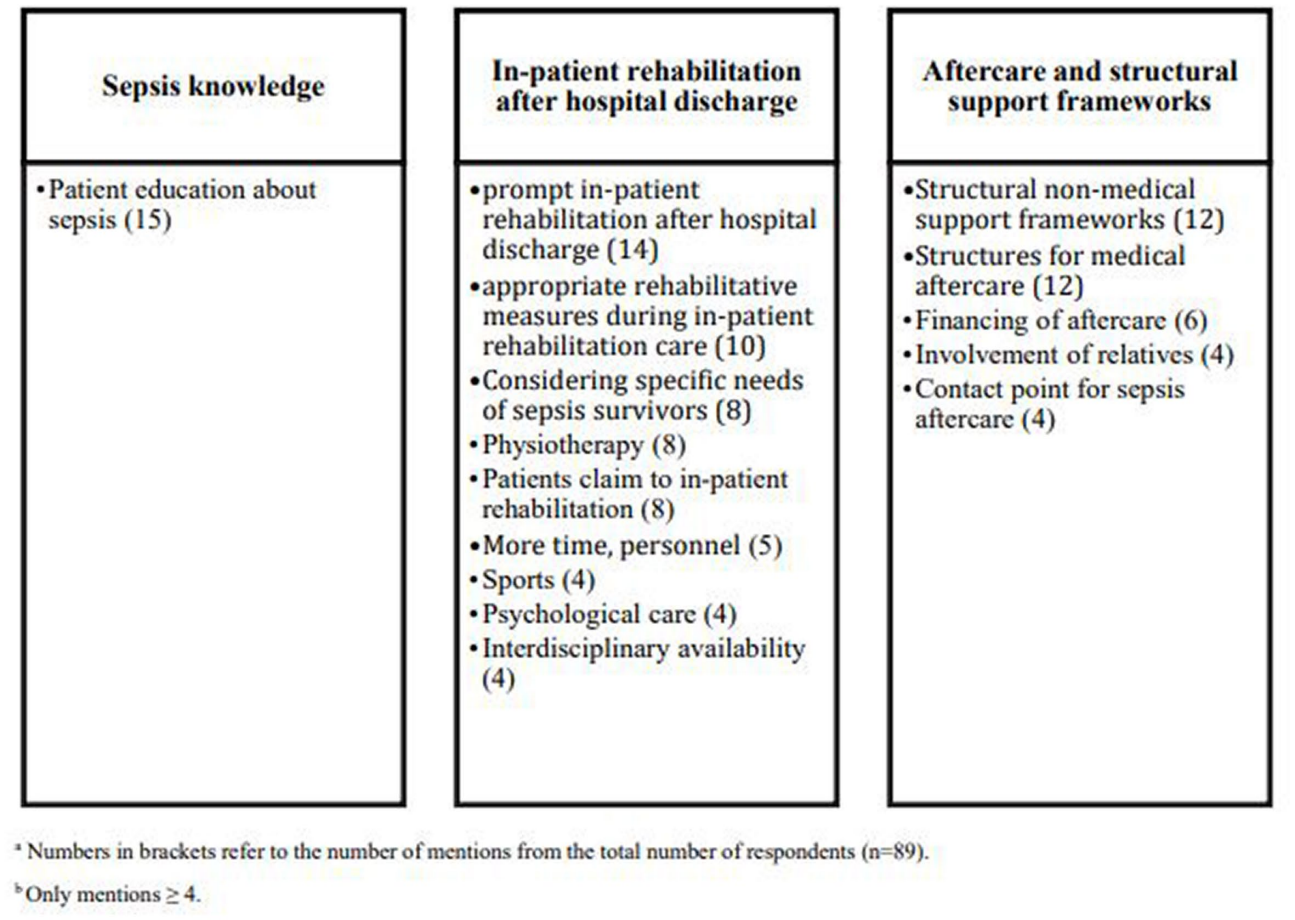
Categorized unmet needs in post–acute rehabilitation and aftercare.

Eighty-Nine patients, their relatives or legal representatives named perceived unmet needs and deficits in rehabilitation and/or aftercare of sepsis. Supplementary Table S1 lists unmet needs as they were mentioned in the interviews including their definitions. They divide into three core categories: unmet needs regarding (a) sepsis knowledge, (b) in-patient rehabilitation after hospital discharge, and (c) aftercare and structural support frameworks (Figure 4). Most of the statements were classified within the category “in-patient rehabilitation after hospital discharge,” and the most frequent mentioned unmet need was patient education about sepsis in the category “sepsis knowledge” Moreover, a prompt further treatment after hospital discharge, the need for out-patient structural non-medical support frameworks, the need of structures for medical aftercare as well as appropriate measures during in-patient rehabilitation care were wished by more than ten interviewees.

## Discussion

This mixed-method study assessed how sepsis survivors experienced rehabilitation and aftercare in Germany. At 6 months after sepsis, 85.0% of survivors had applied for rehabilitation therapy and 70.0% had undergone such therapy at the time of the interview. Given that specific rehabilitation therapies for sepsis sequelae do not exist in Germany, most therapies addressed physical impairments and were provided in neurological facilities although sepsis survivors typically suffer from a range of new cognitive, psychological and physical problems. Less than a quarter of sepsis survivors received specific therapies for their other ailments, including pain, weaning from mechanical ventilation, psychological ailments or fatigue.

Survivors reported overall moderately positive satisfaction with the suitability, extent, and overall results of the rehabilitation, but also perceived deficits in the provision of sepsis-specific in-patient rehabilitation and aftercare and demand better structural support frameworks and patient education. Satisfaction with aftercare is determined by its extent and suitability to a large degree. Given the broad spectrum of possible post-sepsis impairments (2), the high risk of rehospitalization (19) and the life-changing impact of sepsis on survivors and caregivers (20), the lack of structured aftercare and support might prolong recovery (21) and even increase long-term mortality (22). Thus, these results of our study underscore the necessity of improvements in these areas. Our results also have important implications with regard to Covid-19 survivors, as a considerable proportion of Covid-19 patients were affected by sepsis in the acute phase of their disease (23) and long-term impairments after similar and potentially overlapping (24). Therefore, knowledge and experiences from rehabilitation of sepsis survivors can be transferred to COVID-19 survivors (25), which similarly can profit from multidisciplinary rehabilitation for cognitive, psychological and physical impairments (24, 26).

The deficits perceived by survivors are in accordance with an international survey among 1731 sepsis survivors, in which respondents reported their dissatisfaction with the provision of support services in their sepsis and post-sepsis care, particularly with the lack of psychological counseling, education related to post-sepsis symptoms and social services support (6). Similarly, a qualitative study among sepsis survivors and their caregivers in the UK and the US found that the lack of awareness of post-sepsis symptoms and barriers in the access of health care providers and ancillary services after discharge are major challenges perceived (20). These deficits may arise from lacking awareness of the needs of sepsis survivors (20) and the fact that comprehensive post-acute structures are missing, including education, assessment, and rehabilitation facilities. To date, evidence on effective sepsis-specific aftercare is still scarce and isolated strategies examined in previous research, such as post-intensive care ambulances or case-management approaches, did not prove effective to improve psychological impairments or health-related quality of life in sepsis survivors (27, 28). Effective case-management alone did not achieve a change in utilized rehabilitation measures probably because of the lack of suitable measures (27). However, in line with the most recent research, survivors may benefit from structured post-sepsis bundles in outpatient care (22), such as optimization of medication, screening for common impairments and preventable causes of deterioration, and advanced care planning, and post-acute inpatient rehabilitation (29). Moreover, patients may also benefit from an increased provision of follow-up care in existing health care structures.

Our results expand the current knowledge on the use and satisfaction with rehabilitation. Although the rehabilitation system in Germany is unique for instance by the fact that most rehabilitation therapies are conducted in specialized inpatient facilities (30), our study also generates insights valuable for other countries and rehabilitation systems. On the one hand, sepsis survivors suffer from diverse and often overlapping new cognitive, psychological and physical impairments after the acute disease, which require multidisciplinary as well as specific rehabilitation therapies. Rehabilitation tailored toward physical impairments, such as neurological rehabilitation, may therefore not sufficiently address the rehabilitation needs of sepsis survivors. On the other hand, if patients received rehabilitation, subjective patient satisfaction with the rehabilitation was high, even if the rehabilitation only addressed some of the survivors’ impairments. A survey study amongst patients that had received inpatient rehabilitation in Germany found that the general atmosphere (admission procedures, accommodation, catering, service, organization and nursing care) at the rehabilitation facility was the most important contributor to patient satisfaction, followed by the success of the rehabilitation and the quality of the medical care received (31). Considering these findings, high satisfaction perceived by sepsis survivors may be driven by other factors beside the concordance with post-sepsis impairments, which warrants further investigation to better understand contributors to patient satisfaction with post-sepsis rehabilitation and care.

The strengths of our study include the survivor cohort which was identified by a complete screening of ICU patients of two tertiary care hospitals and the Mid-German Sepsis Cohort. By using a mixed-method approach, we were able to complement findings from closed questions with specific perspectives of sepsis and caregivers. Our results deepen the understanding of post-sepsis aftercare and can help to inform patients and families, caregivers, medical professionals and health policy makers on the needs of sepsis survivors regarding rehabilitation, aftercare and patient education.

However, the following limitations need to be considered. First, our study only included patients, relatives, caregiver and legal guardians, who gave informed consent for participation (around one third of eligible sepsis survivors). This may have introduced a selection bias toward patients with less severe sepsis manifestations (12) and limits the generalizability of results. It may also be a possible explanation for the considerably higher proportion of survivors that received post-acute inpatient rehabilitation in our study (70.0%) compared to the findings of a population-based health claims study in Germany (5.5% with discharge to rehabilitation). Above that, our study included ICU-treated sepsis survivors, why our data cannot be generalized to all sepsis survivors including survivors after sepsis treated on regular wards. Second, we assessed the concordance of rehabilitation therapies and new impairments by assessment of self-reported sepsis sequelae, thus we cannot rule out a certain information bias (self-reporting bias). Third, we lack information on the underlying diseases of patients, that may have caused the initial acute care hospitalization and may have contributed to the choice of rehabilitation facilities (e.g., heart attack – cardiopulmonary rehabilitation). Fourth, our study was partly conducted during the COVID-19 outbreak, which may have influenced the use and availability of rehabilitation therapies and aftercare. Fifth, our study did not include a control group, thus we cannot conclude if our observations are sepsis-specific. Further observational studies are needed to investigate to which degree the needs of sepsis survivors differ from other survivors of critical illness or other acute, life-threatening illnesses such as stroke and myocardial infarction.

## Conclusion

Sepsis-specific, interdisciplinary rehabilitation and aftercare, structural support and education may be important measures to improve survivors’ satisfaction with post-sepsis rehabilitation and aftercare. As prerequisite, effective elements of structured post-sepsis aftercare need to be defined and necessary structures within the health system established.

## Data availability statement

The raw data supporting the conclusions of this article will be made available by the authors, without undue reservation.

## Ethics statement

The studies involving human participants were reviewed and approved by Institutional Review Boards of the Friedrich-Schiller University Jena (2018-1223-Bef) and Charité Universitätsmedizin Berlin (EA4/060/19). The patients/participants provided their written informed consent to participate in this study.

## Author contributions

CF-S, CH, and SB supervised the study and designed the questionnaire. AB, H-SB, ME, LH, SS, and KS conducted the interviews and gathered the data. SB, CM-K, and SS analyzed the data. CF-S, SB, CM-K, and CH drafted the manuscript. All authors revised the work for important intellectual content and gave final approval of the version to be published.

## Funding

The study was funded by the German Innovations Fund of the Federal Joint Committee in Germany (G-BA) (grant number: 01VSF17010). The MSC was funded by the German Ministry of Education and Research (BMBF No. 01EO1002 and 01EO1502) and by the Rudolf Presl GmbH & Co, Kreischa, Germany. The funder did not influence the design and conduct of the study; collection, management, analysis, and interpretation of the data; preparation, review, or approval of the manuscript; and decision to submit the manuscript for publication.

## Conflict of interest

The authors declare that the research was conducted in the absence of any commercial or financial relationships that could be construed as a potential conflict of interest.

## Publisher’s note

All claims expressed in this article are solely those of the authors and do not necessarily represent those of their affiliated organizations, or those of the publisher, the editors and the reviewers. Any product that may be evaluated in this article, or claim that may be made by its manufacturer, is not guaranteed or endorsed by the publisher.

## References

[1] Iwashyna TJ Ely EW Smith DM Langa KM. Long-term cognitive impairment and functional disability among survivors of severe sepsis. JAMA. (2010) 304:1787–94. doi: 10.1001/jama.2010.1553, PMID: 20978258 PMC3345288

[2] Prescott HC Angus DC. Enhancing recovery from sepsis: a review. JAMA. (2018) 319:62–75. doi: 10.1001/jama.2017.17687, PMID: 29297082 PMC5839473

[3] Fleischmann-Struzek C Rose N Freytag A Spoden M Prescott HC Schettler A. Epidemiology and costs of Postsepsis morbidity, nursing care dependency, and mortality in Germany, 2013 to 2017. JAMA Netw Open. (2021) 4:e2134290. doi: 10.1001/jamanetworkopen.2021.34290, PMID: 34767025 PMC8590172

[4] Poulsen JB Moller K Kehlet H Perner A. Long-term physical outcome in patients with septic shock. Acta Anaesthesiol Scand. (2009) 53:724–30. doi: 10.1111/j.1399-6576.2009.01921.x 19388891

[5] Prescott HC Iwashyna TJ Blackwood B Calandra T Chlan LL Choong K. Understanding and enhancing sepsis survivorship. Priorities for research and practice. Am J Respir Crit Care Med. (2019) 200:972–81. doi: 10.1164/rccm.201812-2383CP, PMID: 31161771 PMC6794113

[6] Huang CY Daniels R Lembo A Hartog C O’Brien J Heymann T. Life after sepsis: an international survey of survivors to understand the post-sepsis syndrome. Int J Qual Health Care. (2019) 31:191–8. doi: 10.1093/intqhc/mzy137, PMID: 29924325

[7] World Health Organization. (2017). World health assembly 70, resolution 70.7.: improving the prevention, diagnosis and clinical management of sepsis. Available at: http://apps.who.int/gb/ebwha/pdf_files/WHA70/A70_R7-en.pdf (Accessed September 16, 2022).

[8] Konig C Matt B Kortgen A Turnbull AE Hartog CS. What matters most to sepsis survivors: a qualitative analysis to identify specific health-related quality of life domains. Qual Life Res. (2019) 28:637–47. doi: 10.1007/s11136-018-2028-8, PMID: 30350257 PMC6414230

[9] Blumel M Spranger A Achstetter K Maresso A Busse R. Germany: health system review. Health Syst Transit. (2020) 22:1–272. 34232120

[10] Bone RC Balk RA Cerra FB Dellinger RP Fein AM Knaus WA. Definitions for sepsis and organ failure and guidelines for the use of innovative therapies in sepsis. The ACCP/SCCM consensus conference committee. American College of Chest Physicians/Society of Critical Care Medicine. Chest. (1992) 101:1644–55. doi: 10.1378/chest.101.6.1644, PMID: 1303622

[11] Scherag A Hartog C Fleischmann C Ouart D Hoffmann F König C. A patient cohort on long-term sequelae of sepsis survivors - the mid-German sepsis cohort (MSC) study protocol. BMJ Open. (in press) 7:e016827. doi: 10.1136/bmjopen-2017-016827 PMC5623441 28838900

[12] Fleischmann-Struzek C Kesselmeier M Ouart D Hartog CS Bauer M Bercker S. Mid-German sepsis cohort (MSC): a prospective observational study of sepsis survivorship. BMJ Open. (2021) 11:e043352. doi: 10.1136/bmjopen-2020-043352, PMID: 33737430 PMC7978081

[13] Rabin R de Charro F. EQ-5D: a measure of health status from the EuroQol group. Ann Med. (2001) 33:337–43. doi: 10.3109/07853890109002087, PMID: 11491192

[14] R Core Team. (2022). R: A language and environment for statistical computing. (version 4.1.2). Available at: https://www.R-project.org/ (Accessed September 16, 2022).

[15] Rosseel Y. Lavaan: an R package for structural equation modeling. J Stat Softw. (2012) 48:1–36. doi: 10.18637/jss.v048.i02

[16] Schafer JL Graham JW. Missing data: our view of the state of the art. Psychol Methods. (2002) 7:147–77. doi: 10.1037/1082-989X.7.2.147 12090408

[17] Mayring P. Qualitative Inhaltsanalyse. 69 469. Weinheim: Beltz Verlagsgruppe (2015).

[18] VERBI. MAXQDA 2020 [Computer Software]. Berlin, Germany: VERBI Software (2019).

[19] Prescott HC Langa KM Iwashyna TJ. Readmission diagnoses after hospitalization for severe sepsis and other acute medical conditions. JAMA. (2015) 313:1055–7. doi: 10.1001/jama.2015.1410, PMID: 25756444 PMC4760618

[20] Gallop KH Kerr CE Nixon A Verdian L Barney JB Beale RJ. A qualitative investigation of patients' and caregivers' experiences of severe sepsis*. Crit Care Med. (2015) 43:296–307. doi: 10.1097/CCM.0000000000000613, PMID: 25251757

[21] Apitzsch S Larsson L Larsson AK Linder A. The physical and mental impact of surviving sepsis - a qualitative study of experiences and perceptions among a Swedish sample. Arch Public Health. (2021) 79:66. doi: 10.1186/s13690-021-00585-5, PMID: 33933171 PMC8088073

[22] Taylor SP Chou SH Sierra MF Shuman TP McWilliams AD Taylor BT. Association between adherence to recommended care and outcomes for adult survivors of sepsis. Ann Am Thorac Soc. (2020) 17:89–97. doi: 10.1513/AnnalsATS.201907-514OC, PMID: 31644304 PMC6944350

[23] Karakike E Giamarellos-Bourboulis EJ Kyprianou M Fleischmann-Struzek C Pletz MW Netea MG. Coronavirus disease 2019 as cause of viral sepsis: a systematic review and meta-analysis. Crit Care Med. (2021) Publish Ahead of Print:2042–57. doi: 10.1097/CCM.0000000000005195, PMID: 34259663 PMC8594513

[24] Nakanishi N Liu K Kawakami D Kawai Y Morisawa T Nishida T. Post-intensive care syndrome and its new challenges in coronavirus disease 2019 (COVID-19) pandemic: a review of recent advances and perspectives. J Clin Med. (2021) 10:870. doi: 10.3390/jcm10173870, PMID: 34501316 PMC8432235

[25] Prescott HC Girard TD. Recovery from severe COVID-19: leveraging the lessons of survival from sepsis. JAMA. (2020) 324:739–40. doi: 10.1001/jama.2020.14103 32777028

[26] Everaerts S Heyns A Langer D Beyens H Hermans G Troosters T. COVID-19 recovery: benefits of multidisciplinary respiratory rehabilitation. BMJ open. Respir Res. (2021) 8:e000837. doi: 10.1136/bmjresp-2020-000837 PMC8423511 34489236

[27] Schmidt K Worrack S Von Korff M Davydow D Brunkhorst F Ehlert U. Effect of a primary care management intervention on mental health-related quality of life among survivors of sepsis: a randomized clinical trial. JAMA. (2016) 315:2703–11. doi: 10.1001/jama.2016.7207, PMID: 27367877 PMC5122319

[28] Schofield-Robinson OJ Lewis SR Smith AF McPeake J Alderson P. Follow-up services for improving long-term outcomes in intensive care unit (ICU) survivors. Cochrane Database Syst Rev. (2018) 11:CD012701. doi: 10.1002/14651858.CD012701.pub2 30388297 PMC6517170

[29] Rahmel T Schmitz S Nowak H Schepanek K Bergmann L Halberstadt P. Long-term mortality and outcome in hospital survivors of septic shock, sepsis, and severe infections: the importance of aftercare. PLoS One. (2020) 15:e0228952. doi: 10.1371/journal.pone.0228952, PMID: 32050005 PMC7015408

[30] Mittag O Welti F. Comparison of medical rehabilitation in various European countries and the impact of European law on rehabilitation practice in Germany. Bundesgesundheitsblatt Gesundheitsforschung Gesundheitsschutz. (2017) 60:378–85. doi: 10.1007/s00103-017-2516-y, PMID: 28224186

[31] Haase I Lehnert-Batar A Schupp W Gerling J Kladny B. Factors contributing to patient satisfaction with medical rehabilitation in German hospitals. Int J Rehabil Res. (2006) 29:289–94. doi: 10.1097/MRR.0b013e328010b9cc, PMID: 17106344

