# Supplementary material for: Sepsis survivors and caregivers perspectives on post–acute rehabilitation and aftercare in the first year after sepsis in Germany
Source: Front Med (Lausanne). 2023 Apr 11;10:1137027. doi: 10.3389/fmed.2023.1137027 (PMC10126403; doi:10.3389/fmed.2023.1137027)
Supplement: Supplementary file 1 [file Data_Sheet_1.docx]

**Sepsis Survivors and Caregivers Perspectives on Post–Acute Rehabilitation and Aftercare in the First Year After Sepsis in Germany**

Authors

Sebastian Born^1,2^, PhD, Claudia Matthäus-Krämer^1,2^, PhD, Anna Bichmann^3^, MD, Hannah-Sophia Boltz^1,2^, Marlene Esch^3^, MD, M. Sc., Luisa Heydt^1,2^, M. Sc., Stefan Sell, M. Sc.^1,2^, Kathleen Streich^3^, Scherag André^2,4^, PhD, Konrad Reinhart^3^, MD, Christiane S. Hartog^2,5^, MD*, Carolin Fleischmann-Struzek^1,2^, MD*

^1^ Institute of Infectious Diseases and Infection Control, Jena University Hospital, Jena, Germany

^2^ Center for Sepsis Control and Care, Jena University Hospital, Jena, Germany

^3^ Charité-Universitätsmedizin Berlin, Department of Anesthesiology and Operative Intensive Care, Berlin, Germany

^4^ Institute of Medical Statistics, Computer and Data Sciences, Jena University Hospital, Jena, Germany

^5^ Klinik Bavaria, Kreischa, Germany

* contributed equally

**Corresponding author**

Dr. Carolin Fleischmann-Struzek
Stoystraße 3, 07743 Jena
E-Mail: Carolin.Fleischmann@med.uni-jena.de

**Supplement 1**

*Sepsis Criteria*

| Item | Definition |
| --- | --- |
| Sepsis | Presence of infection (I.) AND at least one acute organ dysfunction (II.) |
| Septic shock | Presence of infection (I.) AND hypotension despite sufficient volume substitution (without other cardiogenic or hypovolemic causes) (III.) |
| with |  |
| 1. Infection | Microbiologically proven or clinically suspected |
| 1. Organ dysfunction | - Acute encephalopathy (impaired vigilance, disorientation, agitation, delirium) - Relative or absolute thrombocytopenia (decrease of >30% within 24 hours or number of platelets ≤100.000/mm^3^ (without other cause)) - Arterial hypoxemia (PaO_2_ ≤10 kPa (≤75 mmHg) under ambient air or PaO_2_/FiO_2_-ratio ≤33 kPa (≤250 mmHg) with oxygenation) - Renal dysfunction (diuresis ≤0,5 ml/kg/h for at least 2 hours despite sufficient volume substitution and/or increase of serum creatinine > 2× above locally common reference) - Metabolic acidosis (base excess ≤-5 mmol/l or blood lactate > 1,5× above locally common reference) |
| 1. Hypotension | - Systolic arterial blood pressure ≤90 mmHg for at least 1 hour, and mean arterial blood pressure ≤65 mmHg, respectively   OR   - Need of vasopressor support to raise systolic arterial blood pressure ≥90 mmHg and mean arterial blood pressure ≥65 mmHg, respectively |

*Note*. From “Mid-German Sepsis Cohort (MSC): a prospective observational study of sepsis survivorship,” by C. Fleischmann-Struzek, 2021, *BMJ Open*, *11:e043352. doi: 10.1136/bmjopen-2020-043352*. CC BY-NC.

**Table S1: Open-ended questioned unmet needs for improved care after sepsis**

| **Codes** | **Code definitions** | **Number of mentions (n=89)^a b c^** |  |
| --- | --- | --- | --- |
| **In your view - were there deficits or needs regarding rehabilitation and/or aftercare of sepsis?**  **→ no, yes, I don't know**  **→ if "yes": What would you have wished for?**  **→ open-ended question** | | | |
| Patient education about sepsis | The interviewees reported the need for more detailed patient education regarding the disease, treatment options as well as long-term consequences of sepsis. | **15** |  |
| Prompt in-patient rehabilitation after hospital discharge | The respondents mentioned their wish for in-patient rehabilitation care immediately after hospital discharge. | **14** |  |
| Structural non-medical support frameworks | The need of structural non-medical support after discharge, i.e. patient transport, support during application processes e.g. for remedies and rehabilitation was described. | **12** |  |
| Structures for medical aftercare | The interviewees described the need of interdisciplinary structures for medical aftercare, e.g. treatment by medical specialists. | **12** |  |
| Appropriate rehabilitative measures during in-patient rehabilitation care (suitability, focus, extent) | The wish for more appropriate rehabilitative measures was described. | **10** |  |
| Considering specific needs of sepsis survivors | The respondents mentioned, that specific needs of sepsis survivors, i.e. individual conditions of different kinds of patients, should be considered. | **8** |  |
| Physiotherapy | The interviewees reported, that there were opportunities for improvements regarding physical therapy during in-patient rehabilitation and/or aftercare. | **8** |  |
| Patients claim to in-patient rehabilitation | Patients wished that they have a claim to in-patient rehabilitation. | **8** |  |
| Financing of aftercare | The need of financial support of aftercare was described by the participants. | **6** |  |
| More time, personnel during in-patient rehabilitation | The patients mentioned, that there should be more medical staff available for patient care during in-patient rehabilitation. | **5** |  |
| Sports during in-patient rehabilitation | More sportive activities should be provided during in-patient rehabilitation. | **4** |  |
| Involvement of relatives | Relatives should be considered / involved in disease management processes. | **4** |  |
| Contact point for sepsis aftercare | A contact point for sepsis aftercare would be helpful. | **4** |  |
| Psychological care | Psychological care was mentioned as very important. | **4** |  |
| Interdisciplinary availability | To treat comorbidities, medical staff from other disciplines was considered necessary during in-patient rehabilitation. | **4** |  |

**^a^** Intercoder reliability 59%.

**^b^** If one interviewee named more than one complemented deficit or wish, only one was counted.

**^c^** Only mentions ≥ 4 reported.

**Supplement 2: English translation of the interview questions for sepsis survivors and/or caregivers after 6 and 12 months post-sepsis**

Where was the interview performed? (by telephone, at home, outpatient clinic)

If not performed, what was the reason (patient died, patient/relative could not be reached, current interview was denied, consent was withdrawn, other reasons)

Who answered (patient, relative [ i.e. spouse/partner, child, other relative, other], both patient and relative, age and gender of answering relative

Interview date, time and name of the interviewer

*If the interview is conducted with a relative, please rephrase the following questions or statements appropriately.*

**Demographic information**

Age and gender of sepsis survivor

Highest educational degree of sepsis survivor

Where are you located at the present (at home, rehabilitation facility, nursing home, nursing home with intensive care/long-term ventilation, hospital ward, ICU)

How are you cared for at home at present (not dependent on care/support of others, nursing care by examined nurses, care by partner/spouse - other family members - friends or acquaintances – others)

**Employment, nursing care dependency**

Employed before hospitalization (yes, no)

Nursing care level before your hospital stay (yes, no)

Do you currently have a nursing care level (yes, no)

**Rehabilitation and follow-up care**

In the following, I would like to ask you some questions about your rehabilitation and follow-up care concerning your sepsis illness.

Have you applied for medical rehabilitation? (yes, no)

If yes, how is the actual status of your request? (denied, approved, in process)

If yes, for which kind of impairment have you requested medical rehabilitation? (physical limitations [e.g. limited mobility, coordination disorder, paralysis]), cognitive impairment [loss of memory, concentration, or intellectual capacity]), mental problems [depression, anxiety], acute or chronic pain, impairment of activities of daily living, weaning from longterm ventilation, other, none)

Have you undergone rehabilitation since your hospital discharge? (yes, no)

If you underwent inpatient rehabilitation, please state name and focus (neurological, cardiological, cancer, orthopedic, other, don’t know)

If you underwent outpatient rehabilitation, please state name of the facility and focus (neurological, cardiological, cancer, orthopedic, other, don’t know)

Have you had outpatient therapies after hospital discharge – if yes, please state the kind of therapy (speech therapy, physiotherapy, occupational therapy, functional exercises, psychotherapy, pain therapy, wound management, memory training, others)

If yes, please state duration in days

In your opinion, has there been a lack of medical care regarding rehabilitation and follow-up care after sepsis? (yes, no, don’t know)

If yes, what would you have wished for? (………………………)

**Satisfaction with medical rehabilitation**

*This module only applies if the interviewee has had medical rehabilitation.*

In the following, I would like to ask you some questions about the fit and scope of your medical rehabilitation.

Which kind of longterm impairment(s) were treated by your medical rehabiliation? (physical limitations [e.g. limited mobility, coordination disorder, paralysis]), cognitive impairment [loss of memory, concentration, or intellectual capacity]), mental problems [depression, anxiety], acute or chronic pain, impairment of activities of daily living, weaning from longterm ventilation, other, none)

What kind of therapy/ies have you had during your medical rehabilitation? (speech therapy, physiotherapy, occupational therapy, functional exercises, psychotherapy, pain therapy, wound management, memory training, others)

Answer the following questions according to the specified longterm effects specified above (physicial limitations, cognitive limitations, psychological problems) and overall.

The rehabilitation therapy matched my impairments (disagree – disagree somewhat – agree somewhat – agree)

The scope of the rehabilitation therapy was sufficient (disagree – disagree somewhat – agree somewhat – agree)

I was satisfied with the outcome of rehabilitation therapy (disagree – disagree somewhat – agree somewhat – agree)

If you did not receive rehabiliation therapies – for which impairment would you have wished therapy? (physical limitations [e.g. limited mobility, coordination disorder, paralysis]), cognitive impairment [loss of memory, concentration, or intellectual capacity]), mental problems [depression, anxiety], acute or chronic pain, impairment of activities of daily living, weaning from longterm ventilation, other, none)
